# Supplementary material for: Metagenomic binning with assembly graph embeddings
Source: Bioinformatics. 2022 Aug 16;38(19):4481–7. doi: 10.1093/bioinformatics/btac557 (PMC9525014; doi:10.1093/bioinformatics/btac557)
Supplement: btac557_supplementary_data [file btac557_supplementary_data.zip › amber outputs.zip!/amber outputs/simHC/genome/vamb_edges/heatmap_simHC+.pdf]

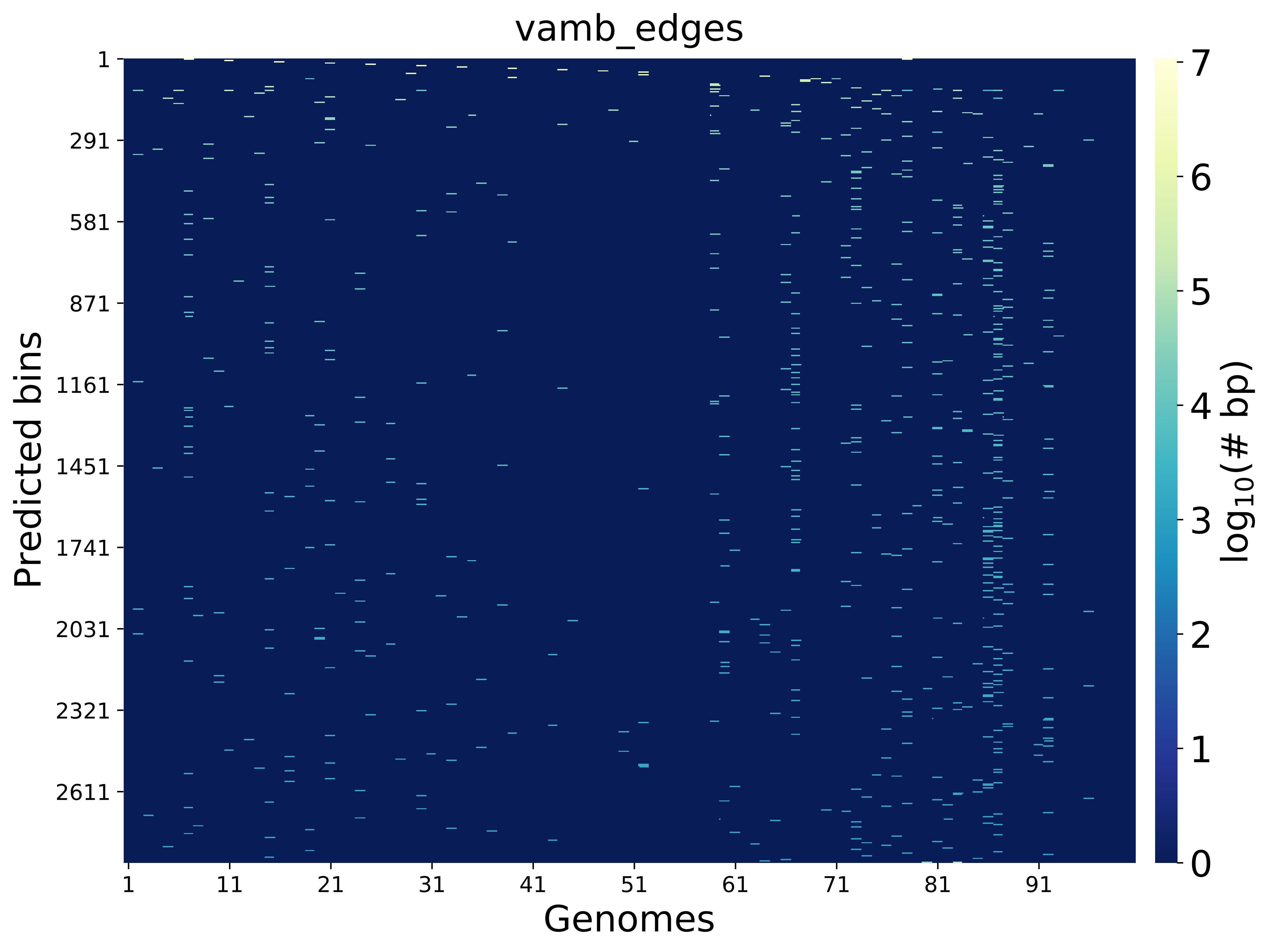

vamb_edges
Predicted bins
1
291
581
871
1161
1451
1741
2031
2321
2611
Genomes
1
11
21
31
41
51
61
71
81
91
log₁₀(# bp)
0
1
2
3
4
5
6
7
